# Recent Advances in Physical Processing Techniques to Enhance the Resistant Starch Content in Foods: A Review

**DOI:** 10.3390/foods13172770

**Published:** 2024-08-30

**Authors:** Muhammad Adil Farooq, Jianmei Yu

**Affiliations:** 1 Institute of Food Science and Technology, Khwaja Fareed University of Engineering and Information Technology, Rahimyar Khan 64200, Pakistan; adil.farooq@kfueit.edu.pk; 2 Department of Family and Consumer Sciences, North Carolina A&T State University, 1601 East Market Street, Greensboro, NC 27411, USA

**Keywords:** resistant starch, physical modification techniques, crystalline structure, granular structure, digestibility, functionality

## Abstract

The physical modification of starch to produce resistant starch (RS) is a viable strategy for the glycemic index (GI) lowering of foods and functionality improvement in starchy food products. RS cannot be digested in the small intestine but can be fermented in the colon to produce short-chain fatty acids rather than being broken down by human digestive enzymes into glucose. This provides major health advantages, like better blood sugar regulation, weight control, and a lower chance of chronic illnesses. This article provides a concise review of the recent developments in physical starch modification techniques, including annealing, extrusion, high-pressure processing, radiation, and heat–moisture treatment. Specifically, the focus of this paper is on the alteration of the crystalline structure of starch caused by the heat–moisture treatment and annealing and its impact on the resistance of starch to enzymatic hydrolysis, as well as the granular structure and molecular arrangement of starch caused by extrusion and high-pressure processing, and the depolymerization and crosslinking that results from radiation. The impacts of these alterations on starch’s textural qualities, stability, and shelf life are also examined. This review demonstrates how physically modified resistant starch can be used as a flexible food ingredient with both functional and health benefits. These methods are economically and ecologically sustainable since they successfully raise the RS content and improve its functional characteristics without the need for chemical reagents. The thorough analysis of these methods and how they affect the structural characteristics and health advantages of RS emphasizes the material’s potential as an essential component in the creation of functional foods that satisfy contemporary dietary and health requirements.

## 1. Introduction

Resistant starch (RS) can be defined as a type of starch that is not digested in the small intestine; instead, it is fermented by gut microbes in the large intestine and produces short-chain fatty acids, such as acetate, butyrate, and propionate. Regular starch is broken down into glucose by digestive enzymes in the gastrointestinal tract (GIT) and is absorbed in the small intestine, whereas RS has similar properties to dietary fiber. Resistance to digestion can be due to starch’s structure, which makes its access to digestive enzymes difficult [1]. Examples of resistant starch-rich foods include unripe bananas, legumes, whole grains, nuts, and cooked and cooled starchy meals, such as rice and potatoes. RS is divided into five categories based on its source and the processing that it undergoes.

RS1: Physically inaccessible starch, which is found in whole-milled or partly milled grains and seeds.

RS2: Starch that is found naturally in raw potatoes and unripe bananas.

RS3: Retrograded starch, prepared by chilling cooked starchy items.

RS4: Chemically modified starch or crosslinked starch.

RS5: Starch that is formed when amylose makes a complex with lipids.

Resistant starch is an interesting topic in food technology and nutrition because of its ability to resist digestion in the upper GI tract [2]. The health advantages of RS are its alteration of gut microbiome and metabolism. Mainly, it can act as a prebiotic, promoting the growth of probiotics in the gut. As a result, it improves digestion, the immune system, and, ultimately, gut health. This is beneficial for alleviating symptoms of constipation and irritable bowel syndrome (IBS). RS can also be beneficial in the management of blood sugar levels by slowing the breakdown of carbohydrates into simple sugar and subsequent absorption into the bloodstream, which ultimately reduces the risk of type 2 diabetes [3]. Since it keeps hunger in control and reduces calorie consumption, it can be related to weight management and increasing satiety [4]. Moreover, the production of short-chain fatty acids as a product of the fermentation of resistant starch in the colon, such as butyrate, can reduce the risk of developing chronic illnesses such as colorectal cancer because of its anti-inflammatory effects. In addition, resistant starch helps in mineral absorption, mainly with calcium and magnesium, which are beneficial for bone health. Furthermore, it can improve cardiovascular health because of its cholesterol-lowering properties. People who add RS to their diet can benefit from a wide range of advantages for their overall well-being [5].

Because of the health advantages of RS and its techno-functional attributes in the food system, it is desirable to change simple starch into resistant starch physically. This goal is achieved by modifying its structure, making it less susceptible to digestive enzymes and thus increasing its resistance. The molecular structure of starch can be modified by physical, chemical, enzymatic, and biotechnological methods. The physical treatments include heat–moisture treatment, retrogradation, high-pressure processing, radio-thermal methods, annealing, ultrasonification, and pulsed electric field treatments. These modifications improve the textural properties, stability, and shelf life and increase the dietary fiber content and, ultimately, the nutritional value of the products [5].

This review explains the sources, structural properties, and manufacturing processes of resistant starch in detail and includes both the natural forms and physical modification processes. It also elaborates on the health advantages of resistant starches as well as the technical uses and benefits for food systems, highlighting functional meals that can satisfy dietary and health standards. Through this in-depth analysis, this review seeks to highlight the importance of resistant starch in nutrition and food science, offering insightful information for future studies and useful applications in the food sector.

## 2. Modification of Resistant Starch

Starches, in their raw form, have several unwanted properties that make them inappropriate for most uses; hence, they are modified to form a variety of alterations to boost their favorable traits and/or reduce their flaws. Modification is the process of altering the characteristics of natural starch; modified starches attain an increased paste clarity, paste and gel textures, film formation, and adhesiveness but result in decreased retrogradation, paste-gelling tendencies, and gel syneresis [6].

**Figure 1:**
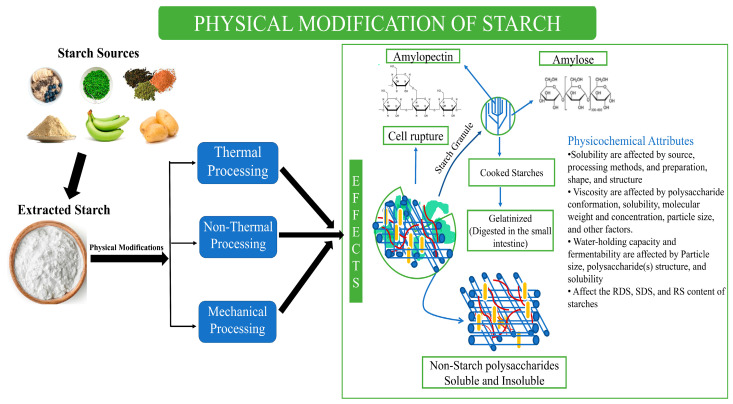
Sources of RS, types of physical modifications, and the effect of modification on starch structures and properties.

The modification methods that are currently in use and their effects on starches are shown in Figure 1. Physical treatments are conducted by thermal and non-thermal treatments and by mechanical methods. To rearrange the amylose-to-amylopectin ratio, and the length of the chain, thermal treatment is used [7]. Thermal treatments include heat–moisture treatment (HMT), annealing, and roasting. Non-thermal treatments include high-pressure, irradiation, microwave, and pulsed electric field treatments. However, mechanical physical modification includes milling, grinding, and extrusion [7]. Another method is chemical modification, which is the insertion of new functional groups on a starch backbone that can provide unique properties to starches. The methods of chemical modification include hydrolysis, oxidation, esterification, and grafting [8,9]. The enzymatic modification of starch can target the amylose chain length, molecular weight, and amylopectin. Enzymatic modification cannot improve the swelling ability of starch granules, but it can be used for disintegration [9].

### 2.1. Physical Modification

#### 2.1.1. Mechanical Processing

##### Milling

The milling of cereals results in damaged starch granules. During milling operations, shear and stress are applied to cereal grains, which alters the morphology and functional properties of starch, including its solubility, swelling power (SP), heat stability, retrogradation, pasting behavior, and digestibility [10,11,12,13]. Ball milling is an advancement in the milling process, which is an economical and environmentally friendly process that uses mechanical activities (compression, abrasion, collision, and pressure) to change and decrease starch to fine granules. It constitutes one of the physical alteration strategies used to lower the overall crystallinity and enhance the digestibility of starch. Moreover, milling has shown a reduction in phenols, flavonoids, and dietary fiber content, while with an increased degree of milling, whiteness and structural changes in the aroma occurred. The milling speed and time increase the swelling power and solubility of amylose (AM) [14]. The research findings show that milling alters the physical structure of brown rice, which increases the contents of rapidly digestible starch (RDF) and decreases the slowly digestible starch (SDF) [15].

The nutritional value of starch is dependent on its digestibility. On the basis of digestibility, starch is classified as rapidly digestible starch (RDS), slowly digestible starch (SDS), or resistance starch (RS) [16]. The damaged starches due to milling have higher water absorption and hydrolysis rates as compared to the unmilled starches of wheat, potato, maize, and rice [17,18,19]. An increase in the level of damage to starch granules can lead to an increase in digestibility. A study reported that jet milling damaged rice starch and resulted in an increase in rapidly and slowly digestible starch contents but decreased the RS content [18]. Moreover, a positive correlation was found between the degree of damaged starch in isolated wheat starch and RDS [19]. Starch granules damaged by milling have a higher surface area and large hollows inside the granules, which facilitate more interaction between enzymes and starch. Additionally, the distinctive granular structure of milled damaged starch facilitates easier water diffusion and enzyme penetration, which is crucial for enzymatic digestion [20]. Milling also disrupted the crystalline structure of starch and resulted in the formation of more amorphous regions, which made it more accessible to digestive enzymes to hydrolyze the starch [21]. Milling affects the molecular composition of starch. It was reported that an increased degree of polishing resulted in a high amylose content in waxy rice starch and that starch with a high amylose content and single helices needed more time to be hydrolyzed to restrict the amylolytic enzymes from penetration [22,23].

##### Grinding

Grinding is a beneficial technique for altering and processing various materials. This treatment typically results in a decreased crystal structure and particle size, which may facilitate processes such as physical alloying and powdered cold jointing. The superfine grinding of corn straw results in a powder of up to 9–16 μm, and the addition of these microparticles to corn starch-based films results in corn-straw-starch-based films with better mechanical properties, such as improved cellulose content and creep-resistance properties [24]. The grinding technique demonstrates the changes in starch granules that produce a high-quality RS. Research on starch acetate production found that wet grinding mung bean starch for 4 h significantly changed the mechanochemical properties of the corn starch granules, and the efficiency of starch acetate formation was increased from 70 to 85% by the grinding technique [25]. A high-quality modified starch with improved swelling power and solubility was obtained from mung bean with 12 h of grinding followed by acetification [26]. The grinding technique has also shifted towards wet grinding to avoid dusting problems during dry grinding. Wet grinding increases grinding homogeneity, and water promotes the motility of starch molecules and acts as a plasticizer. Wet grinding also promoted the formation of starch–lipid complexes, and the resistant starch content of starch reached a maximum of 35.61% after 30 min of wet grinding. The wet grinding technique improves starch’s swelling power and thermal stability [27].

##### Extrusion

Extrusion is known as a thermal processing technique in which an uncooked mass, such as cereal foods, is subjected to high heat, high pressure, and shear forces. The extrusion technique is widely used in the food sector for its superior product quality, variety, low cost, high production, energy efficiency, and environmental friendliness. During the extrusion process, the starch undergoes structural changes, including starch gelatinization, degradation, and retrogradation [28], mainly influencing the nutritional and physiochemical characteristics. These structural changes can modify the properties of proteins, carbohydrates, and other substances, which results in large variations in the digestibility of starch [29,30].

The extrusion cooking process (ECP) is a feasible and environmentally friendly alternate for producing RS. It was reported that the ECT produced 1.15 g/100 g of RS at 233 rpm, 127 °C, and with an 18% moisture content. The ECP, along with the addition of citric acid addition, increased the amount of RS by 71% in comparison to native starch [31]. The digestibility of extruded starch can vary depending on the physical characteristics of the raw materials, such as the source of the starch granule, the material particle size, and the content of dietary fiber, protein, and lipids, as well as the ratio of amylose to amylopectin [30]. The starch digestibility of different genotypes of beans has been tested and proven to be affected because of the diverse amylopectin content and different starch granule sizes. The starch granule type and crystallinity have been reported to vary depending on the plant source. It was reported that the RS content in extruded corn starch was 2.5%, which is less than extruded kidney beans (16.3%) and field peas (15.6%) [30]. The different c-type crystalline structures of starch cause these variations, as the native kidney bean and field pea both have a mixture of orthorhombic and hexagonal crystals, while corn starch only has orthorhombic crystals. Hexagonal crystals can be identified by the multiple branches that are gathered in the amorphous regions, and are characterized to be rigid and can tolerate enzymatic hydrolysis as compared to the orthorhombic crystals. On the other hand, the particle’s size is considered an important aspect in determining starch digestibility after extrusion [32]. For example, finely milled sorghum and barley grains provide better digestibility because of the increased surface area for interactions with the digestive enzymes as compared to the coarsely milled grains after extrusion [32]. Also, the rate of water penetration differs with the particle size during starch gelatinization, which ultimately affects digestibility [32]. In the extrusion process, the formed RS is due to the change in amylose molecules [33]. Thus, the amylose content plays a major role in determining starch digestibility after extrusion, as starch with a high amylose content forms more RS as compared to the waxy starches [34,35].

Also, starch’s digestibility is affected by the lipid content. The roles of lipids include lubricating, reducing the required mechanical force during the extrusion process, and degrading the starch macromolecules [36]. The digestibility of the extruded starches is affected by the amount of amylose–lipid complexes formed between amylose and the intrinsic and extrinsic lipids, and this defines RS5 [37]. This is attributed to the reduced amylose solubility and interference of V-type crystals in enzyme attacks [38]. The interaction between amylose and lipids during the extrusion process has been studied and proven in previous studies, and the number of formed complexes depends upon the characteristics of both starch and the amylose content in food [39,40]. In addition, not every fatty acid can form complexes with amylose. For instance, monoglycerides and free fatty acids can form a complex easily, while triglycerides cannot because of their large size and inability to enter the starch spiral to form a stable helix [40]. It is certain that, the more the amylose content, the shorter the fatty acid chain and the more complexes that can be formed [41]. On the other hand, the chances of amylopectin forming a complex is much smaller because of the short side chain and steric hindrance of amylopectin [42]. Also, the degree of saturation of the fatty acids affects the amylose–lipid complex; saturated fatty acids are preferred over unsaturated fatty acids because the rigid molecular structure of unsaturated fatty acids prevents access to the amylose helix [43].

Protein affects starch digestibility by forming a complex around the starch granule and preventing a reaction with the digestive enzymes [44]. For example, beans have a slow starch digestion rate because of the high protein content in them [45]. Also, protein hinders the swelling and gelatinization of starch granules during the extrusion process [46].

Furthermore, the dietary fibers present in the raw material can affect starch digestibility. For example, when processing barley extrudates and adding tomato and grape pomace, starch digestion is reduced because of the entrapped starch within the protein, fiber, and starch network structure [47]. Apple pomace also lowers the hydration level during starch gelatinization, which ultimately reduces starch digestibility [48].

### 2.2. Thermal Treatments

#### 2.2.1. Heat–Moisture Treatment (HMT)

A physical technique, heat–moisture treatment (HMT), uses controlled temperatures and moisture contents to alter starch. The integrity of the starch granules is preserved since this method prevents gelatinization [49]. This technique has been extensively researched by scientists using a wide range of botanical sources. Specific contents of amylose and amylopectin have been measured in relation to variables such as time, temperature, moisture content, and drying apparatus [50]. The HMT is a highly acclaimed hydrothermal approach for physical modification that involves exposing starch to a reduced moisture content (below 35%) at a higher temperature (80–140 °C) for certain periods of time [51]. It is well known that the HMT increases the levels of SDS and RS and enhances the nutritional value of starch-based products [52]. Some of the specific elements linked to the effect of HMT on starch digestibility are the amounts of amylose and amylopectin, the linkages and structures for these macromolecules, the processing parameters, the botanical origin of the starch, as well as properties like the crystallinity and granule size of the starch granules [49]. As starch digestibility is related to overall wellness, particularly for those with diabetes, the modification of starch digestibility by HMT is crucial for consumers [53]. The study of the digestibility of HMT rice starches shows that rice starch exhibits small, medium, and large granules in contrast with various other starch sources; the last is related to poor in vitro rice starch digestibility. Following HMT mutation, the starches of rice, wheat, and potatoes were more susceptible to α-amylase, while there was a drop in RDS and a rise in the SDS and RS ratios in the starches of maize, peas, and lentils [53]. The RS content is impacted by the temperature as well as other aspects of food preparation and storage.

The study by Liu et al. [54] found that ANN and HMT treatments of corn starch altered the characteristics of starches, including gelatinization and retrogradation, amylose-to-amylopectin ratio, and crystalline structure, which are necessary for the enzymatic susceptibility of starch. Generally, a lower RDS concentration and higher SDS and RS indicate less digestibility. This occurs when structural changes in the starch granules tighten them up and make it harder for the digestive enzymes to break them down [55]. The HMT of potatoes at 100 and 110 °C for two hours using moisture contents of 30 and 35% encouraged a decrease in the RDS and a rise in the SDS and RS [56]. The RS values increased by 95% and 88%, respectively, at 100 °C and 120 °C, with a 35% moisture content. This result suggests the formation of a complex structure that limits the ability of enzymes to break apart starch molecules. Greater effects on RDS and SDS were observed at the lowest temperature (100 °C) and maximum moisture (35%) exposure. The disparate results that the authors present for several parameters clarify that starch responds differently to the temperature and moisture content, which could potentially open up possibilities for new applications. Liu et al. [54] heated modified maize starch at 110 °C for 16 h and varied the moisture percentages of 20, 25, 30, and 35% using HMT. The modification actually caused the SDS and RS levels to rise, but the RDS level to fall. At the optimal moisture content (35%) employed in HMT, the lowest RDS value and highest SDS and RS values were observed. We hope that these research findings will support the practical application of this physically altered starch for preventative measure against chronic illness.

To assess the impact of HMT on digestibility, Chung et al. [56] modified germinated brown rice kernels (with a moisture content of 30% at 100 °C for 1 h). The ongoing refinement of the granule’s crystalline structure and amylose retrogradation account for the decrease in RDS and SDS levels and the rise in RS level. However, Zhao et al. [57] found that when mung bean starch underwent repeated heat–moisture treatment (RHMT) (with a 30% moisture content at 120 °C for 2, 3, 4, 5, and 6 h) or HMT (with a 30% moisture content at 120 °C for 2, 4, 6, 8, 10, and 12 h), the RS content reduced but the SDS and RDS contents increased. The authors explain that a rise in enzyme susceptibility may cause RS to convert to SDS. Decreased bonding between starch molecule chains would result in changes to the crystalline structure and disintegration of starch granules. The higher relative crystallinity of starches after RHMT may be the cause of the higher SDS and RDS values. As a result, RHMT promoted the formation of a starch that is easier to digest and may be utilized in meals for a fast energy supply.

The amount of amylose in starches and the degree to which HMT affects this starch’s characteristics can differ. Wang et al. [58] used HMT for two hours at 120 °C and with moisture levels of 20, 25, and 30% to modify common and high-amylose maize starches. As the moisture content rose, the RDS values dropped. The largest decrease in RDS was observed at a 30% moisture content for both starches (24% for common corn starch and 43% for high-amylose corn starch). The SDS and RS contents increased dramatically in comparison to the untreated starches. At a 30% moisture content, the RS increased by almost 1000% with a high amylose percentage as a result of a larger degree of disorganization followed by the rearrangement of high-amylose starch caused by HMT. This research shows that HMT is a great method for converting high-amylose starch into RS.

#### 2.2.2. Annealing

Annealing (ANN) is a thermal treatment of starch in the presence of excess water for an extended period of time. This physical modification method can raise the amount of RS because of the way starch chains interact and reassemble. ANN alters the physicochemical characteristics of starch without altering its granular structure. The temperature, heating period, and starch-to-moisture ratio are important variables influencing the RS yield and must be regulated throughout the hydrothermal treatments. ANN includes heating starch granules at a temperature below the gelatinization temperature (GT) and above the glass transition temperature (Tg) when they are either over-hydrated (76% *w*/*w*) or have an intermediate water content (40% *w*/*w*) [59]. The most typical ANN-induced alterations are as follows: (1) increased gelatinization temperatures and gelatinization enthalpy concomitant with a narrowed gelatinization temperature range; (2) increased long-range crystalline structure (relative crystallinity); (3) increased short-range crystalline structure; (4) reduced swelling capability, solubility, and paste viscosity; and (5) reduced digestibility [60]. The study by Wang et al. [61] revealed that the structures of wheat, potato, and yam starches were little affected by ANN, but their GT rose, although the enthalpy change was little affected by ANN. The effects of annealing on the paste viscosity of wheat starch varied with the annealing temperature. The paste viscosity rose when annealed at 30 and 40 °C, but it significantly decreased at 50 °C [61]. It consistently strengthens starch molecules, which enhances the paste viscosity, stability, and in vitro enzymatic digestion while causing only minor structural alterations [62]. Nonetheless, annealing’s effect on the RS content differs based on the botanical source. Starch’s crystal type and relative crystallinity are two crucial properties that affect this process [63]. Annealing is generally beneficial for improving the structures of crystalline and amorphous lamellae and rearranging the starch chains without compromising the granule shape [64].

Annealing is a widely employed technique that modifies the molecular integrity of starch by modifying its qualities, such as relative crystallinity, water absorption capacity, and pasting properties. Additionally, the starch digestibility that results from these hydrothermal treatments directly affects consumer health outcomes because the RDS concentration is decreased while RS and SDS are developed during annealing [65]. The primary cause of these alterations in starch digestibility is the disturbance of starch structure, which makes starch molecular chains more accessible to amylolytic enzymes. Additionally, this modification changes the crystalline structure of starch, improving its digestibility [66]. Song et al. [67] found an increase in the RDS and SDS contents but a decrease in the RS content in potatoes and sweet potatoes after annealing. The reason behind this is that amylolytic enzymes attack more amorphous regions. Furthermore, during the partial gelatinization of annealed starch, the α-helical structure was lost, which increased the SDS content but lowered the RS content. However, the data collected from different studies showed no effect of annealing on the overall digestibility of starches [54,55,67,68,69]. In order to reduce the digestibility and improve the SDS and RS contents, there is a need to apply annealing appropriately based on the specific type of starch.

#### 2.2.3. Roasting

Roasting is a food processing method that uses the heating principle to cook food uniformly and improve digestibility, palatability, and sensory qualities while achieving the desired structural changes to the food matrix [70]. Roasting alters the food product’s structure and quality, affecting the functional, physical, chemical, and nutritional properties. During this process, an increase in porosity softens the kernels and disrupts the endosperm structure [71]. The dry and hot air creates a low moisture–vapor pressure gradient, which results in moisture evaporation and pushes the internal moisture of the food to migrate towards the surface [72]. Roasting can increase the grain shelf life by inactivating the enzymes and reducing moisture. In addition, roasting also changes the properties of grain components, such as starch, particularly when there is a lot of moisture present. Typically, roasting is conducted at temperatures between 100 and 220 °C, with a modified moisture content of 10–15%, and for a duration of between 10 and 60 min [73]. Roasting disintegrated the grain structure and led to particle agglomeration. Microwave, infrared hot air, and superheated steam, as well as the Revtech roaster and forced convection continuous tumble (FCCT) roasting, are some of the roasting methods that have been increasingly rising to prominence for successfully roasting various meals without sacrificing their nutritional value [70]. Scanning electron microscopy (SEM), differential scanning calorimetry (DSC), Fourier-transform infrared (FTIR), and Raman spectroscopy have been used to analyze barley, rye, triticale, oat, sorghum, and millet flours that had been subjected to roasting or extrusion treatment to identify the structural changes within the starch and protein molecules caused by roasting [74].

Roasting can have different effects on starch digestibility depending on the type of grain and the roasting temperature. It has been reported that dry heat treatment, as compared to wet heat treatment, results in a higher RS content in a variety of foods such as cereals, tubers, and legumes [75]. It is found that high moisture levels during thermal treatment facilitate amylose leaching from starch granules, which increases the solubility and susceptibility of starch [76]. On the other hand, roasting damages the structure of starch and partially gelatinizes and then retrogrades the starch, which positively affects RS development [77]. The impact of various dry-heating procedures on the production of RS in rice and millet has been investigated by optimizing the duration and temperature combinations for a maximal resistant starch yield in convectional electromagnetic heat treatments. This offers great promise for the invention of low-cost traditional techniques for producing high-quality food items with high concentrations of RS, which can then be utilized to formulate a variety of functional foods. Pan roasting, fluid-bed drying, tray drying, and convection microwave heating techniques were used to maximize the RS output in rice and barnyard millet. Convection microwave heat-treated samples had the highest level of RS for both rice and millet [78].

#### 2.2.4. Microwave

Microwave electromagnetic waves, ranging from 300 MHz to 300 GHz with wavelengths of 1 m to 1 mm, are widely used in the food industry for thermal heating at frequencies like 915 MHz or 2450 MHz. The regulatory capability of the rapid oscillation of the microwave electric field is vital for the effective management and oversight of irradiated food production [79]. Dielectric properties, influenced by factors such as microwave frequency, food composition, density, and temperature, determine how effectively the foods absorb and convert microwave energy into heat. Starch-based materials exhibit distinct dielectric properties due to the varying moisture contents, mineral ion concentrations, and processing parameters like temperature and porosity, which affect their response to microwave treatment [80]. Microwave technology provides a dynamic way to increase the amount of RS in food items [81]. Electromagnetic radiation is applied during microwave processing, usually at frequencies between 300 MHz and 300 GHz. By interacting with the polar chemicals and water molecules in food, this radiation causes molecular friction and fast oscillation, which produces heat [82,83].

The electromagnetic field present in microwave ovens operates at a high frequency that increases the temperature of the medium, thus inducing the nonionizing energy [84]. RS3 has been previously documented as having been prepared in a system containing an ample amount of water by microwave cooking, while other studies have modified dry starch with a lower water content through microwave irradiation (microwave–HMT). The multistage process of starch gelatinization is irreversible and occurs when starch granules are heated in a large amount of water. Starch granules become swollen, the native crystalline structure melts, the birefringence is lost, and eventually, the starch is solubilized. Braşoveanu and Nemţanu have studied the physicochemical, functional, and structural changes of starch samples when treated using microwave–HMT [85]. It was observed that the mechanism of action involves the following: (i) the water molecules are subjected to the dielectric relaxation phenomenon; (ii) the rapid increase in temperature causes the building up of high pressure in the starch granules; and as a result, (iii) the granules burst, which increases the volume; and ultimately (iv) the granules are degraded. Yang et al. [86] have reported that major changes occur in the internal chains of the amorphous region during the early stages of starch granules when subjected to microwave–HMT, whereas, in the later stages, the external chains of the crystalline region become disrupted.

Starch gelatinization rapidly occurs when heating the starch granule with a large amount of water, and this can be achieved by microwave–HMT. For this reason, the use of microwaves has gained researchers’ attention for producing RS3 from multiple botanical sources. Microwave irradiation helps in achieving the desired heat level in a short time span, which improves the starch structure and yields an excess amount of RS3 [87]. Other advantages of microwave irradiation include the production of free radicles, which help in hydrolyzing the glycosidic bonds in starch granules and the fermentation of large granules into smaller ones [88].

According to HNMR data, the breakdown of the α-1,6-glycosidic bond happens easily as compared to the α-1,4-glycosidic bond [86]. Deka and Sit [89] have reported that 20.08% more amylose content was yielded when taro starch was cooked in the microwave as compared to the 13.90% value for native starch. This can be attributed to the rapidly absorbed energy by the amylopectin chains that lead to its breakdown due to heating in a microwave. Zeng et al. [90] found that the molar mass distribution of RS samples decreased upon cooking in a microwave, as evaluated by SEC-MALLS-RI. Also, Fan et al. [87] documented that the use of SAXS during microwave cooking changed the lamellar structure. The double-helix structure of amylopectin is compressed and ordered into a crystalline structure at the beginning of microwave heating at 40 °C. Zeng et al. [90] reported that the production of RS from lotus seed during microwave cooking was operative as compared to autoclaving. Palav and Seetharaman [91] confirmed these findings. This can be due to the fact that the higher microwave temperature did not cause the amyloses to spread out completely from the native starch granule; thus, the chances of formation of a double-helix during retrogradation and the formation of RS3 are reduced. A comparative study by Zeng et al. [90] concluded that the RS particles prepared by microwave exhibited a smaller and smoother surface, as observed by SEM, compared to the RS particles prepared by autoclaving, whereas it was confirmed by the solid-state ^13^C NMR that microwaves had a marginal influence on amylose dissolution, for which the amorphous components were intensified upon microwave cooking as compared to the RS treated by other methods such as ultrasonic-autoclaving or autoclaving. Zeng et al. [90] have stated that when a 15% starch suspension was cooked in the microwave, the sub-crystalline structure of RS increased as compared to other samples prepared by autoclaving; this explains the importance of sufficient water use for the formation of primary crystalline structures. 

One of the benefits of cooking native starch in excess water in a microwave is the crystalline structure changing from a C- to a B-type as compared to the microwave–HMT that amended the starch crystal structure from a B-pattern to an A-pattern or from a B- to a C-polymorph. Another study observed a pronounced difference in the birefringence that occurred earlier than gelatinization during microwave cooking because of the vibrating water molecules [92]. The SAXS results have also demonstrated the reasons behind complete gelatinization, including the crystal structure loss earlier in microwave cooking as compared to conventional heating because of the immediate increase in temperature and the vibrating motion. 

A study using microwave irradiation to produce RS3 observed that microwave cooking enhanced the formation of RS3 after RS2 degradation under minimal intensity [93]. Also, the same study found that retrogradation in a microwave oven at 95 °C for 24 h enhanced the contact of amylose chains and the formation of a double-helix structure. 

Maximizing RS yields major benefits when microwave technology is applied. Not only does microwave processing improve the nutritional profiles by lowering the glycemic index and raising the dietary fiber content, but it also has other benefits, like reduced processing times and nutrient loss and improved product stability. This approach takes into account consumer preferences for less processed, healthier foods and is adaptable to a broad variety of food products, such as cereals, vegetables, and baked goods [82]. The microwave treatment (600 W, 6 min) was examined to assess its effects on the physicochemical properties and the in vitro digestibility of the starch of three sorghum varieties, Longmiliang No. 1 (LML1), Longza No. 13 (LZ13), and Hongnuo (HN). Following the treatment, there was a significant increase (3.72–7.45 g/100 g) in the insoluble dietary fiber, soluble dietary fiber, and total dietary fiber contents in all sorghum types (*p* < 0.05). The starch granules exhibited rougher surfaces, and there was an increase in the pasting temperature and time, along with a decrease in the pasting viscosity and transition enthalpy. The microwave treatment also reduced the in vitro digestibility of sorghum starch by 3.21–6.61%, decreased RDS by 5.88–9.24%, increased SDS and RS by 4.63–6.65% and 1.03%–2.41%, respectively, and lowered the hydrolysis index (HI) and glycemic index (GI) by 4.98–5.74% and 2.73–3.15%, respectively [94].

### 2.3. Non-Thermal Processing

#### 2.3.1. High-Pressure Processing (HPP)

High-pressure processing (HPP) is a non-thermal approach that has been used as a food preservation method to inactivate microorganisms and enzymes in foods. HPP has been used more often by researchers to increase the amount of RS in foods [95]. This process works on the basis that food products are exposed to high hydrostatic pressure (usually between 100 and 800 MPa), which causes structural changes in the starch molecules and other ingredients without the need for heat [96]. HPP applies high hydrostatic pressure to food items using specialized equipment. A high-pressure vessel, commonly referred to as an autoclave or pressure chamber and usually made of sturdy materials like stainless steel, is the main piece of equipment used in HPP. Through molecular structure disruption, HPP facilitates the formation of RS, which resembles the properties of dietary fiber and provides improved nutritional benefits like improved colon health, decreased glycemic response, and increased satiety [97].

HPP has been the subject of recent research aimed at increasing the levels of RS in different food products. Studies have investigated the use of HPP in purees of fruits and vegetables, rice, bread, bakery goods, potatoes, beans, and lentils, as well as dairy products like yogurt [98]. Through the application of high hydrostatic pressure, scientists have changed the structure of starch molecules in these foods to produce RS. A study reported a decrease in RDS content and an increase in the levels of SDS and RS when HPP techniques were applied to sorghum starch [99]. The in vitro digestibility of potato starch decreased by 10–15% after a two-step HPP treatment (step one at 400 MPa and step two at 600 MPa), followed by retrogradation at 4 °C for 7 days [100]. Another study reported an increase in thehydrolysis rate (466%) in sorghum starch [101]. In contrast, a small increase was observed in HPP-treated maize starch [102,103]. The reason behind this is that starch formed a more compact structure after HPP. Moreover, no positive correlation has been found between the magnitude of pressure and the increase in RS. For example, the increased RS contents in corn, pea, and rice starches were observed when the starches were treated at a pressure of 200 and 400 MPa, respectively [104,105,106]. On the other hand, some studies reported a higher hydrolysis rate and lower RS content when treated with HPP [107,108,109,110]. For example, HPP resulted in an increase in the enzyme hydrolysis rate by 129% in buckwheat starch [110]. Moreover, a decrease in the RS content was observed in rice starch when treated at 600 MPa [111]. This might be due to the partial or complete gelatinization of starch caused by high pressure, which resulted in structurally damaged starch molecules that could be degraded by the digestive enzyme at an increased hydrolysis rate [98].

The digestibility of starches was not affected by changing the processing time and temperature. For example, a study reported no significant difference in the hydrolysis rates of amaranth, quinoa, and wheat starch when HPP treatments were conducted at 40 °C and 60 °C [112]. The same study found no increase in the RS content when the HPP treatment was 30 min and 5 min at the same pressure [112]. Moreover, a suitable combination of HPP and other methods may be appropriate to achieve better results.

#### 2.3.2. Irradiation

Ionizing radiation, such as gamma rays or electron beams, has been used to improve food safety and extend the shelf life and may also be applied to induce particular structural alterations in starch molecules as part of the radiation-based physical processing approaches for improving RS in food products [113]. There is a strong correlation between gamma radiation and a decrease in the starch swelling index, as well as the apparent amylose content peak, setback, trough, and final viscosities. Modified starch is a useful substitute ingredient for frozen foods because gamma irradiation improves the starch’s functional properties, such as reduced retrogradation and gelatinization enthalpy [114]. Gamma (γ) irradiation offers several advantages as a physical therapy, including its great efficiency, low cost, environmental friendliness, and no significant temperature increase throughout the treatment process. Radiation-induced starch breakdown is repeatable and quantifiable, requiring no particular temperature or environmental controls. The impact of radiation on starch has garnered much attention in the past 10 years [115]. A few recent studies examined how γ-radiation affects the morphology, structure, and physiochemical characteristics of starch obtained from numerous sources, including maize, rice, arrowroot, potato, and amaranth [116,117,118].

Gamma irradiation can be utilized to increase the fraction of RS. It may produce reactive oxygen species, which can lead to molecular alterations and starch fragmentation. Starch depolymerization causes an ongoing decrease in the molecular dimensions of both amylopectin and amylose by spontaneous breakage of glycosidic chains, altering the crystalline and physicochemical properties of starch. There were no visible or substantial alterations in starch granules following irradiation [115].

γ-Radiation can greatly affect the enzyme susceptibility of starch when different doses are implemented. However, contradictions have been observed among the different studies [119,120,121,122,123]. Studies reported an increase in the RS content [119,120] and a decrease in the SDS content of corn, potato, and white bean starches [119,121]. This was due to the variations in starch composition, such as the moisture content and irradiation conditions. For instance, a higher RS content was observed in the corn starch containing 5% moisture upon irradiation, while a lower RS content was detected in corn with 12% moisture [123]. Lee et al. [122] studied the effects of different radiation doses on all corn starches with a 0–70% amylose content. They found a higher RS content when employing 5 KGy and 50 KGy, while 10 KGy led to a decreased RS content. Furthermore, no effect was observed when crosslinked waxy rice was treated by γ-radiation at a dose of up to 100 KGy [121]. During irradiation, several structural and chemical changes occur. At high doses, the γ-ray energy may be absorbed by starch and directly cleave the glycosidic linkage, or the γ-ray may first interact with water molecules, producing free radicals and peroxides, which induce the cleavage of glycosidic linkages [120,122]. Radiation processing, in contrast to traditional thermal processing techniques, works at room temperature, reducing thermal deterioration and maintaining the sensory qualities, nutritional value, and general quality of the treated foods [124].

Processing variables influencing the conversion of regular starch to RS and the integrity of the food include the dosage rate, total dose (measured in kilograys, kGy), and ambient conditions (temperature, humidity). These variables must be carefully regulated. By increasing the amount of dietary fiber in food, this method not only improves its nutritional profile but also lengthens its shelf life by lowering the microbial load, improving food safety, and decreasing food waste. To ensure the safety of irradiated food, ongoing regulatory improvement and strict enforcement are required, including radiation level monitoring. The international alignment of standards is crucial. Despite regulatory efforts, unlabeled irradiated food persists due to unlicensed operations, causing market confusion. However, China faces challenges in adopting certification requirements for irradiation facilities, as mandated by the EU and US. It is vital to enhance regulatory capabilities for the effective management and oversight of irradiated food production and sales and to address the current gaps in certification protocols and health science standards [79].

#### 2.3.3. Ultrasonication

A new environmentally friendly and sustainable way to alter natural starch for enhanced performance is ultrasonication. Ultrasonication treatment generates shear forces, which disrupt the starch structure and break down long starch chains into short chains. This non-thermal processing method promotes the formation of RS by disrupting the crystalline structure of starch, resulting in a change from a V-type to a B-type crystalline pattern [125]. The ultrasonic treatment of starch suspension at 360 kHz for 3 h at room temperature decreased the molecular weight due to the chain dissociation of starch molecules in the suspension [126]. A similar result was reported by Zhu et al. [127], who found that the cluster structure of starch granules was modified by ultrasonic treatment in the presence of excess water (155 W for 30 min at a temperature between 20 °C and 30 °C) and resulted in a decrease in molecular order in crystalline lamellae. The data obtained from the SAXS study showed that granules swelled and loosened because the high ultrasonic power transferred the fractal structure of starch from the surface to the mass fractal. Furthermore, a study reported a decrease in the electron density difference between the crystalline and the amorphous lamellae. These changes, induced by ultrasonication, distort the crystalline regions and increase the amorphous areas of starch molecules [128,129].

By breaking down the crystalline structure of starch granules, ultrasonication treatment makes them more accessible to enzymes and discourages the production of RS [130]. This process is carried out in specialized sonication chambers with ultrasonic transducers that convert the electrical energy into mechanical vibrations within the instrument to break down the crystalline structure of simple starch to produce RS [131]. Further modification of the ultrasonification process is needed to increase the effectiveness of the process.

Regarding nutritional enhancement, RS is a key component because of its similarities to dietary fiber, which is important for better glycemic control and digestive health. Microscopic cavitation bubbles are produced within food matrices by ultrasonication, which uses high-frequency sound waves that are typically between 20 kHz and 80 kHz. Without having a major temperature impact, these quickly collapsing bubbles provide strong localized stresses that break and alter starch molecules [132].

Ultrasonication has provided a versatile option to create functional food and improve nutrition by increasing the amount of RS. For instance, ultrasonication has effectively changed the starch structures of cereals such as barley and quinoa, which increased the amount of RS without substantially affecting the taste or texture. Additionally, ultrasound-treated root vegetables, such as beets and carrots, exhibited beneficial effects on digestive health by enhancing the production of RS [133]. However, it is important to consider the processing conditions, such as the temperature, moisture level, duration of treatment, and varietal differences, as these factors can lead to conflicting outcomes in the studies examining the impact of ultrasonication on RS levels [134]. 

#### 2.3.4. Pulsed Electric Field (PEF)

Pulsed electric field (PEF) processing is a new non-thermal food pasteurization method that uses short bursts of high-voltage electric fields on food to achieve the desired microbial inactivation or structure modification. The PEF technology helped in retaining the basic starch properties, the ratio of amylose and amylopectin, hydrogen bonds, and diffraction patterns [135,136]. Thus, the PEF technology might be used to produce native starch, although extra research to confirm its economic sustainability is required. Corn starch could be improved by PEFs by enhancing the quality of damaged starch, enthalpies, gelatinization temperatures, pseudoplastic behavior, in vitro digestibility, and resistance to deformation [137]. Moreover, a PEF at a lower intensity altered the shape of starch granules without affecting Maltese crosses. However, these Maltese crosses disappeared when a stronger electric field was implemented. Type A and type B starches were more vulnerable to these alterations as compared to the C-type starches. A PEF significantly affects the starch structure, reduces the relative crystallinity, and reduces the gelatinization temperature and enthalpies, viscosity, and pasting temperature. Moreover, a PEF also affects the in vitro digestibility of starches by increasing the RDS content, reducing the SDS content and maintaining the RS content. One study treated different starches, such as wheat, potato, and pea, with a PEF intensity of 2.86 to 8.57 KV/cm and reported a significant increase in the RDS level and a decrease in the SDS content while the RS content remained the same as compared to their counterparts [137]. Similar results were reported by Wu et al. [138] when they treated rice starch (A-type) with a PET intensity from 2.86 to 8.57 kV/cm. On the other hand, another study reported a similar result for waxy starch but found a reduced RS content [139]. Polymorphism damage and morphological changes may occur when starch granules are exposed to PEF treatment. These changes increase the accessibility of enzymes to new and/or more numerous glycosidic linkages and make them more digestible by damaging the starch structure. A PEF also reduces the molecular weight of starch chains. A study reported a decrease in the molecular weight of maize starch chains when it was subjected to an increasing electric field intensity from 30 to 50 kV/cm and a treatment time from 424 to 1272 μs (r^2^ > 0.95) [140]. The result indicates that the electric field intensity has more effect on the molecular weight as compared to time. Amylopectin destabilization may be another cause of the molecular weight reduction of starch chains. A study observed an increase in the relative molecular weight of short and long amylopectin chains when the PEF intensity exceeded 5.71 kV/cm [139]. This might be caused by the breakdown of the amylopectin chain, as suggested by Li et al. [137]. Future studies are needed in order to understand the detailed structural changes observed with amylopectin and how a PEF affects its susceptibility to enzymatic breakdown.

Another important question that arises here is the behavior of modified starches on the human digestive tract, their effect on human health, and their practical applications. A study observed a decrease in potato starch digestibility after 120 min of PEF treatment compared to the untreated starch. The reduction was due to the starch disruption when the starch was treated at 1.1 kV/cm and 50 kJ/kg [141].

Thus, PEF technology might be at the forefront of food processing to provide RS in culinary goods. The PEF technology allows for the conversion of digestible starch into RS by delivering controlled, brief pulses of high-voltage electric fields that electroporate starch granules and cell membranes [142]. PEF treatment transforms starch into resistant starch in grains and cereals, and this will lower the glycemic index of starchy food. Table 1 shows the effects of different processing techniques on the RS contents of foods.

## 3. Differences among Modification Methods and Their Effects on RS

Milling and grinding use mechanical force to reduce the particle size of starch. They are widely employed in food processing to improve the texture and mixing properties of food ingredients [157]. It must be mentioned that by reducing the particle size, the starch granule structure is disrupted, leading to an increased surface area and increasing starch hydrolysis rate by digestive enzymes and ultimately reducing the RS content [17,158,159]. According to previous studies, RS can be transformed into easily digestible starch segments by fine grinding [160,161,162]. The extrusion process is considered a quite complicated process, and it involves the use of high pressures, temperatures, and shear forces to cook the starch and change its shape. This process is employed to make cereals, snacks, and pet foods. It requires a great level of precision and accuracy in temperature, moisture content, screw speed, and feed ingredients [147]. The extrusion process reduces the content of RS and makes it easily digestible because of the gelatinization and dextrinization that occur under high temperatures and shear forces. However, during the extrusion process, retrograded starch (RS3) may be formed upon cooling because of the realignment of the crystalline structure [163]. Therefore, to use extrusion in food processing, the extrusion process must be improved to stabilize the production of digestible starch and RS. 

Studies have proven the use of heat–moisture treatment (HMT) to process functional food products with a high percentage of dietary fibers to enhance the RS content in multiple starch sources, such as maize, wheat, and potato starch [56,164,165,166,167]. HMT is operated at high temperatures (80–120 °C) and a low moisture content (10–30%) for a defined period. This enhances the structural rearrangements of starch granules, thus resulting in resistance to enzymatic breakdown [168]. The advantage of this treatment is that it modifies the starch structure without initiating gelatinization. By this treatment, the ordered crystalline structures that resist amylolytic enzymes are formed. HMT is proven to increase RS2 (resistant starch that stays ungelatinized) and RS3 contents [56,169,170].

Annealing is a thermal process that treats starch at a temperature below its gelatinization temperature and uses a surplus amount of water for a prolonged period. Annealing is performed at a lower temperature and allows flexible molecule movement in the starch granule, unlike HMT, which produces a perfect, stable crystalline structure [150]. By employing this treatment, the RS content is enhanced by rearranging the amorphous structure to a stable crystalline. Because of the compacted internal structure, annealed starches are resistant to enzymatic hydrolysis [171]. The mild flavor that is developed during annealing treatment makes the starch acceptable in various food products. Studies have shown that the annealing process increases the RS content in potato starch, foxtail millet starch, and high-amylose maize [172,173,174].

Roasting exposes starch to a dry heat treatment at high temperatures, causing the starch to gelatinize partially and ultimately retrograde. Through this process, the RS3 content is increased. In this process, the starch granule swells and gelatinizes, partially because of the high temperature. After cooling, the structure becomes crystalline because of the realignment of amylose and amylopectin chains [77]. Roasting can be employed to increase the RS content in legumes and cereals for the production of functional foods with high dietary fiber [75]. Furthermore, roasting enhances the sensory properties of food products [152].

High-pressure processing (HPP) is a non-thermal treatment during which the starch undergoes a very high pressure (up to 600 MPa) for a definite period. HPP treatment changes the structure of the starch granule, which facilitates the formation of crystalline RS because of the stable crystalline structure. According to the studies, starch processed with HPP can produce a higher RS content as compared to unprocessed ones [175,176,177]. The advantages of employing HPP for making functional foods are that the nutritional value and sensory attributes are preserved while a high RS content is produced.

When starch is exposed to ionizing radiations such as γ-rays or electron beams, the crosslinks between starch granules are enhanced, and the structures are modified, making them resistant to digestive enzymes [178]. The produced stable molecular structure from irradiation is less affected by hydrolysis [179]. Researchers have shown that the RS content is increased by irradiating maize starch and potato starch [120,122]. The main point of irradiation is that the dose level must be accurately controlled to produce functional food with a high RS content.

The microwave irradiation of starch in the presence of water increases molecular friction, thus causing rapid heat development. As a result, RS3 is formed because of the partially gelatinized and retrograded starch [180]. When the structural changes occur because of microwave treatment, the RS content is increased, and ultimately, the resistance to enzymatic hydrolysis is increased, too [181]. Studies have reported that a higher RS content was determined from the starch treated with microwaves as compared to that treated with conventional heating [182]. Because of the efficiency and the speed of microwave heating, it is most practically used to make functional food products with high dietary fiber. In addition, microwaves can be employed in food processing lines as a cheaper solution to enhance starch application.

Ultrasonication is a process that uses high-frequency sound waves to cause the starch granules to vibrate and disrupt. The effects of ultrasonication on the RS content depend on the processing conditions. Mild ultrasonication conditions can promote the production of RS3 and promote partial gelatinization and retrogradation [183]. Harsh processing conditions, such as long durations, high temperatures, and strong power, are known to break the crystalline starch structure, thus reducing the RS content [184]. It is crucial to maintain ideal processing parameters to increase the RS content and preserve the properties of starch.

Pulsed electric field (PEF) processing is a non-thermal method that involves the use of short bursts of high electric field voltage on starch. This benefits the modification of the starch granule structure that leads to the formation of a stable, resistant crystalline structure, thus resistant to hydrolysis by enzymes. According to the studies, a high RS content is produced from PEF-treated starch as compared to untreated ones. This benefits the production of functional foods with higher dietary fiber content. The PEF processing conditions must be controlled to enhance the RS content in starchy foods.

Physical treatments employed for changing and altering starch granules offer a variety of methods, and these mechanisms are unique and produce a high content of RS. Milling, grinding, extrusion, and other mechanical treatments increase digestibility by reducing the RS content because of an interrupted granular structure and size reduction. On the other hand, annealing, roasting, heat–moisture treatment, and other thermal treatments increase the RS content and enhance starch’s resistance to enzymatic hydrolysis by promoting structural changes. However, HPP, irradiation, microwaves, PEF, ultrasonication, and other non-thermal treatments showcase the different effects on the RS content, depending on the techniques and treatment conditions used. Structural alignments and crosslinking are some of the mechanisms of RS formation by non-thermal treatments, whereas a reduction in RS content is caused by breaking down the resistant structures.

Improvements in the functional properties of food products rich in RS require the selection of proper methods to reach the highest level of effectiveness. Improved health outcomes can be attained by understanding the different ways in which RS is generated, thus contributing to functional food production. In future studies, different methods used should be optimized to increase the RS content and preserve the nutritional quality of other food components. In addition, studies must highlight the combined effect of multiple treatments to offer promising end results when it comes to the nutritional values and health aspects of resistant starch-based functional food.

## 4. Health Benefits of Resistant Starch

RS has positive effects on managing obesity, diabetes, cardiovascular diseases, and colon health. These health benefits originate from its resistance to hydrolysis and tendency to escape digestion in the upper gastrointestinal tract, which ultimately reduces the absorption of glucose and enhances the concentration of short-chain fatty acids, i.e., butyrate, propionate, and acetate in the colon. Digestive diseases are positively associated with unbalanced diets [185]. The importance of an increased consumption of indigestible carbohydrates can be found in a recent review [186]. RS has half of the calorific value (7 Kj/g) as compared to digestible starch (15.1 Kj/g). Aside from the glycemic response [185], RS can be fermented by colonic microbiota to produce short-chain fatty acids [187]. The prebiotic production of shorter-chain fatty acids makes the environment less liable to develop cancerous tumors. RS may have effects on the prevention of colonic cancer [188], constipation [189], lowering the pH of the colon [190], and levels of ammonia [191].

**Figure 2:**
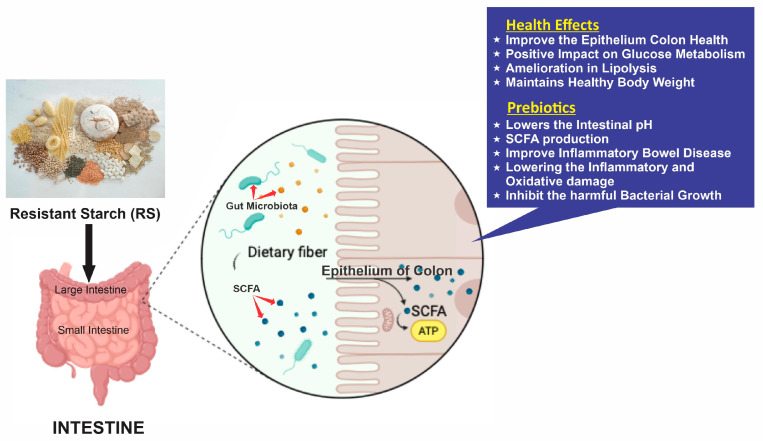
Resistant starch sources, their fermentation by human gut microbiota in large intestine and their health benefits produced.

Intestinal microbes digest the foods containing RS in the large intestine. They have a particularly significant impact on gut health. Various compounds such as hydrogen and methane, along with short-chain fatty acids (SCFAs)—mostly acetic, propionic, and butyric acid—are generated along with valerian, iso-valeric, and iso-butyric, which are present in minute quantities. SFCAs are substances that can lessen the levels of blood triglycerides and cholesterol, supply energy to colon cells, and contribute to sustaining the healthy epithelium of the colon. Furthermore, the fermentation of RS has a positive impact on the glucose utilization and insulin synthesis, leading to a gradual liberation of dextrin in the blood’s circulation. Some research also proposes that the intake of RS has a favorable impact on the receptivity of insulin achieved by decreasing excess fat storage in non-fat tissues and modulating adipocyte development [3]. Reduced postprandial glycemic and insulinemic responses result from RS, which causes a slower metabolism of blood sugar. In addition, RS has been shown in both healthy individuals and people with metabolic syndromes to offer several physiological benefits, including controlling blood lipid levels, enhancing insulin sensitivity, and influencing appetite [192] (Figure 2).

RS can be considered a prebiotic since most types of RS stimulate good microflora and elevate the amounts of overall SCFA in the gut, particularly butyrate, which is advantageous to the host’s health [193]. Enhancing feces size has several advantages, including alleviating constipation and shortening the length of diarrhea. Furthermore, it was discovered that RS2 ingestion helped improve inflammatory bowel disease (IBD) and lower myeloperoxidase activity. RS consumption may inhibit colonic carcinogenesis, according to several animal studies. The way RS interacts with intestinal microbiota is primarily responsible for its positive benefits. The regulation of the host’s metabolic processes, particularly immunological growth, as well as the equilibrium of glucose and insulin, is carried out by their intestinal microbiome. RS acts as a base for probiotic microorganisms to grow as it passes through the small intestinal tract. SCFAs provide several advantages for intestinal health, including lowering inflammatory and oxidative damage, excreting gastrointestinal hormones, and controlling the homeostasis of glucose [194]. As a result, RS functions as a prebiotic to attenuate the glycemic index of meals, leading to reduced absorption of blood sugar in the gastrointestinal system, even in the presence of polyphenols. This action restricts the enzymes responsible for breaking down starch in the cells lining the intestines [195]. RS has the ability to lower the pH in the intestines. This can help suppress the growth of pathogenic bacteria such as *Salmonella* and *E. coli*, which are sensitive to changes in pH. The by-products of RS digestion, especially SCFAs, can affect the release of intestinal hormones, regulate blood sugar levels, reduce inflammation, and minimize oxidative damage, leading to various positive effects on digestive health [194].

RS increases the gut signaling chemicals that regulate hunger, stop fat from accumulating, and aid in maintaining a healthy body weight. The growing research evidence indicates that RS increases satiety, improves tolerance to insulin, decreases lipid metabolism, as well as reduces postprandial blood sugar levels [196]. The potential pathways that contribute to satiety enhancement include longer intestinal transit times for fulfilling macronutrients, decreased lipid absorbance, and increased release of intestinal hormones that regulate hunger. Two main gastrointestinal hormones that control the utilization of energy as well as promote satisfaction via the central nervous system responses are peptide YY (PYY) and glucagon-like peptide 1 (GLP-1). RS may trigger the secretion of these hormones by stimulating intestinal L-cells with SCFAs generated during digestion. It increases the sensation of fullness and decreases calorie consumption [194].

It is evidenced that butyrate, the SCFA raised by RS use, lowers colonic swelling, provides energy to the intestinal epithelium cells, and lowers the possibility of malignancies in the colon [192]. RS may use more extensive fermentation-related processes to provide defensive benefits. The three most prevalent anionic forms of SCFAs produced by intestinal microbial digestion are butyrate, propionate, and acetate. They exert their beneficial impacts by either increasing or decreasing the synthesis of cytokines that trigger inflammation and by either promoting or hindering the entry of cells from the immune system, as they are essential in regulating swelling. Methane, hydrogen, carbon dioxide, and trace quantities of naturally occurring acids like lactate, succinate, and formate, as well as branched SCFAs, are produced during the digestion process of RS in the gastrointestinal tract. Microorganisms, such as *Bifidobacteriaceae* or *Lactobacillaceae*, are developed to decrease irritation [195]. Elevated concentrations of *Bifidobacterium* and *Lactobacillus* species have been experimentally shown to strengthen immunity and prevent the growth of tumor cells. An adequate balance of gut microbiota can have a role in the deactivation and elimination of harmful substances, lessen swelling, or substantially improve immunological reactions [197].

RS has a significant effect on mineral bioavailability. The number of minerals in rats and humans, and their iliac absorption are enhanced by RS [189]. In one study performed, studies summarized in the review of [198] have shown that rat diets rich in RS increased the absorption of calcium, magnesium [186], phosphorus, magnesium [199], zinc, iron, and copper [200]. This effect is limited to calcium only in humans. The increased mineral absorption is because the SCFAs produced by RS fermentation lower the pH in the colon, thus accelerating the conversion of mineral elements into soluble ions that are easily absorbed in the colon [201,202]. Table 2 provides a concise overview of the health benefits associated with different types of RS.

## 5. Applications of Modified Resistant Starch

Many products, such as yogurt, ice cream, cheese, bread, and fermented drinks, contain significant amounts of RS [3]. Each type of RS has its unique technological and physiological functions [210]. When a high temperature and moisture are included in the process, RS3 can be formed while RS1 and RS2 can be destroyed [187]. RS3 formation may be enhanced by cooling of baked product to ambient temperature, while the RS2 content of potato can be increased during refrigeration storage; however, the RS content of cereal can be reduced [188]—starch storage at low temperatures increases in revision [211].

Starch plays a very important role in bread texture and quality [212]. Replacing wheat flour partially with starch leads to a decreased extensibility of the bread. Starch dilutes the gluten and leads to gelatinization by absorbing water and gas formation in the bread so that the bread does not collapse during the cooling process [213]. The removal of gluten from bread is the biggest challenge [212]. The bread quality properties increase while the gap between regular bread and gluten-free bread is filled with a high amount of fiber. The volume of gluten-free bread increased with an 11–15% inclusion of modified starches due to the increase in the number of gas cells and decrease in gas cell size in gluten-free bread [214].

Consumer-required quality attributes of muffins include them being tender, spongy, and soft with a degree of resistance [215]. The addition of RS to the muffin formula did not alter the texture and properties of the product greatly. All types of RS muffins have a denser structure compared with the native starch of muffins [216]. RS2 is less viscous than RS3 [210]. Because of the variety and nutritional quality, cookies are consumed by all populations. RS-enriched cookies have a high amount of dietary fiber and a low in vitro glycemic index [210]. The addition of RS3 and RS4 increased the density but reduced the breaking strength of cookies [217].

Color is one of the most important properties of fried battered products, which represents the time to control frying, and golden brown is one of the most desirable colors [218]. The main intensified color properties come from the milliard reaction (a reaction between sugar and amino acids to produce melanoidin, a compound that gives brown food products) [219] and the caramelizing reaction (a process of browning of sugar). For acceptance by customers, scores are given for color, oil, flavor, and crispiness, and the overall score should be higher than 6 [220]. In one study, the effects of the addition of type 2 and 3 RSs in fried battered products were evaluated. The study concluded that RS3 contributed more to the development of a dark color than RS2 [221]. Therefore, the addition of RS3 may be able to improve the color and nutritional values of the product [199].

Some research has found that pasta enriched with RS4 improves the texture [222]; however, pasta enriched with a 5% bran portion affected the product positively [223]. The addition of RS2 up to 10% had no negative effect on pasta quality. An amount of more than 10% reduced the yellowness and brightness with minimal effects on redness [224]. A 51% addition of RS2 resulted in brighter pasta than any of the other pasta [225]. A substitution with 20% of RS2 does not affect the sensory properties, such as being chewy, rubbery, or slipperiness [185]. One study was conducted by Cervini et al. [226] in which the properties of pasta dough with RS2 and RS3 were investigated on two levels. The addition of more RS to the dough may weaken the dough [225]. The pasta enriched with 11% of RS3 had minimal changes in cooking loss [223] but increased firmness; however, this property was not changed when RS2 was used [185]. Firmness is associated with the content of gluten in pasta. For this reason, a limited addition of RS to pasta should be considered [227].

RS is suitable for snacks because RS has a high fiber content without compromising the quality of the product [3]. It is present in both processed and unprocessed foods. Regarding the nutritional benefits, starch in food can be classified into three categories [228], namely, slowly digestible, rapidly digestible, and resistant starches. As the market lacks good fiber sources for snack products, the new RS presents new categories of good fiber to snack consumers [201].

The application of different types of RS in dairy products as a fat replacer and stabilizer has been researched. In the 1990s, the cheese industry introduced low-fat cheese into the market. For instance, 11–20% of fat is present in mozzarella cheese [229], so it is great to substitute a portion of fat with RS [230]. However, cheese with RS3 as a fat replacement had an increased hardness and fiber content. The effects of RS3 were more than RS2, while RS2 showed greater influence on increased cohesiveness [231]. The smooth and homogenous texture of cheese can be improved by replacing up to 43.2% of fat with RS without a noticeable change to the moisture content [232]. Research conducted by Hajian and colleagues [233] shows that maize RS, xanthan, and Arabic gum as stabilizers in camel’s milk ice cream significantly influenced the physicochemical properties of the product. The sample containing the maximum amount of RS and gum had the least overrun value and viscosity but had the lowest melting rates [234]. The adhesiveness of the ice cream was not affected by the addition of RS. Increasing the amount of xanthan and Arabic gum improved the sensory properties of camel’s milk ice cream [235].

The applications of different resistant starches in various food products and their benefits are listed in Table 3.

## 6. Conclusions

Significant functional and physiological benefits can be obtained from the resistant starch developed by the physical alteration of regular starch. Extrusion, annealing, heat–moisture treatment, and high-pressure processing are some of the techniques that efficiently increase starch’s resistance to digestion and raise the dietary fiber content. These techniques also give starch better textural qualities, stability, and shelf life, which makes it appropriate for a range of food applications. In order to maintain blood sugar levels, promote healthy weight, improve gut health, and lower the risk of chronic diseases, RS is essential. The food sector can create modern meals with high RS through the application of the above-mentioned modification techniques. Food technology advancement is the future of the food industry and increases consumer awareness about sustainability and health impacts. Starch modification is shown to be an active component in improving public health and reaching sustainability objectives.

## 7. Challenges and Future Directions

Rising worries over an environmentally friendly source of RS will drive studies in this area. In recent years, the percentage of studies devoted to the development and implementation of RS by physical methods has grown significantly because most physical methods do not introduce external chemicals into the starch and do not generate waste; they are safe and environmentally friendly. In food, RS is an important discussion topic, as evidenced by the increasing number of articles published in recent years. It will also pique the interest of future scholars. More research is needed to develop RS with improved techno-functional qualities, including solubility, consistency, and thermal resistance. Several commercial RS preparations are currently available, which can be used to improve the dietary fiber content of foods. The health benefits of RS are widely acknowledged. Advanced food processing technologies have resulted in lower RS consumption. Because of its beneficial functional and physiological qualities, commercial RS formulations are increasingly used in processed meals. With the current information on RS, it is extremely difficult to recommend an appropriate amount of RS for consumption for general health advantages, although several figures have been given for specific health benefits. Therefore, more research is required to develop RS formulations with optional properties that can claim specific health benefits, such as enhanced gut health and a lower glycemic response. In addition, contradicting results about the effects of a physical method on the formation of RS were reported by different researchers. Therefore, further research is needed to comprehend the science fully.

## Figures and Tables

**Table 1:** Effect of processing techniques on resistant starch contents of foods.

Processing Technique	Description	Mechanism of Action	Effect on Rs Content	References
Ball milling	Grinding and crushing balls inside a revolving drum.	Particle size reduction and starch amorphization.	Reduces the RS concentration by fracturing starch’s crystalline structure.	[143,144]
Wet grinding	Uses a ball mill where the grinding chamber has been filled with water.	Combination of shear, impact, and attrition forces with water.	Reduces the RS content of starch and encourages gelatinization.	[145,146]
Extrusion (barrel screw extrusion)	Single/twin revolving screw to form and transport material through a die inside a heated barrel.	Combination of heat, pressure, and shear to break down and reorganize starch molecules.	Reduces RS level due to significant granule gelatinization and fragmentation.	[147,148]
Heat–moisture treatment	Heating starch for a predetermined amount of time at a high temperature and with limited moisture.	Modifies the amylose-to-amylopectin ratio and encourages retrogradation.	Increases RS content, increases RS5 and RS3.	[49,149]
Annealing	Heating starch for a prolonged amount of time with a high moisture content.	Enlarges starch granules without losing their integrity with high moisture content.	Rise in RS content. Increases RS3 and RS2.	[63,150]
Roasting	Heating starch at high temperatures with little to no water content.	The starch granules undergo both chemical and physical changes at a high temperature.	Boosts the formation of RS4 and increases the concentration of RS.	[151,152]
Ionizing radiation	γ-ray or electron beam penetrates into food materials at regulated dosages, causing ionization and excitation of molecules.	Causes the depolymerization and crosslinking of starch molecules by rupturing chemical bonds.	Primarily raises RS4.	[115,153]
Ultrasonication	Employs high-frequency sound waves to cure liquid starch.	Causes starch granules to break apart and partially gelatinize.	Boosting the production of RS3 can also improve the formation of RS4.	[154,155]
Pulsed electric field	Exposing starch in a liquid media to brief high-voltage bursts.	Enhances the absorption of water and makes starch modification easier.	Improves retrogradation and encourages structural improvements to raise RS content.	[138,156]

**Table 2:** Health benefits related to resistant starch.

Type of Resistant Starch	Health Benefits	Description	References
RS2, RS3	Digestive health	It acts as a prebiotic, bypasses digestion, avoids spiking glucose, and reaches the large intestine’s gut to feed good bacteria.	[3]
RS3, RS4	Blood sugar control	Lowers postprandial blood glucose and insulin levels and improves metabolic health.	[203]
RS2, RS3	Weight management	Increases satiety and reduces overall calorie intake, aids in weight loss by reshaping the gut microbiota.	[196]
RS3	Colon health	Produces short-chain fatty acids like butyrate, which have anti-inflammatory properties and reduce the risk of colorectal cancer.	[204]
RS2, RS3	Cholesterol reduction	Lowers LDL and total cholesterol levels, which improves cardiovascular health.	[2]
RS2, RS3	Improved mineral absorption	Enhances the absorption of minerals such as calcium and magnesium in the colon.	[189]
RS3, RS4	Enhanced immunity	Modulates immune response by promoting microbial-derived metabolites and dampening neutrophil recruitment.	[205]
RS3, RS4	Reduced inflammation	Lowers systemic inflammation; beneficial for conditions like inflammatory bowel disease (IBD).	[206]
RS3	Gut barrier function	Strengthens gut barrier integrity and prevents leaky gut syndrome.	[207]
RS2, RS3	Improved insulin sensitivity	Enhances insulin sensitivity, reducing the risk of type 2 diabetes.	[208]
RS2, RS3	Weight loss and satiety	Promotes feelings of fullness, reducing overall calorie intake.	[209]

**Table 3:** Applications of resistant starches in different food products and their benefits.

Applications	Food Products	Types of Resistant Starch	Benefits	References
Baked Goods	Bread, Muffins, Cookies, Cakes	RS2, RS3, RS4	Improved texture, increased dietary fiber content, enhanced shelf life, reduced the glycemic index of products.	[200,236,237]
Pasta and Noodles	Spaghetti, Rice Noodles	RS3, RS4	Lower glycemic index, improved gut health, increased insoluble dietary fiber, increased satiety.	[224,238,239]
Snacks	Chips, Crackers	RS2, RS3	Reduced calorie content, high in fiber, nutrient-dense, lower salt and sugar, prevents non-communicable diseases, suitable alternative for gluten-intolerant people, improved digestibility.	[240]
Dairy Products	Yogurt, Cheese, Ice Cream	RS2, RS4	Prebiotic effects, enhanced creaminess, increased iron and fiber levels, increased viscosity, and sensory properties.	[189,200]
Meat Products	Sausages, Meatballs	RS4, RS5	Improved texture, fat replacement, and increased fiber content; acts as a prebiotic.	[200,241]
Breakfast Cereals	Cornflakes, Granola	RS3, RS4	Higher fiber content, lower glycemic response, reduced risks of colon cancer, coronary heart disease, and enhanced crunchiness.	[223]
Beverages	Smoothies, Meal Replacement Drinks	RS2, RS3	Improved satiety, prebiotic effects, lower glycemic index, good for gut health, regulates glucose homeostasis.	[242]
Gluten-Free Products	Gluten-Free Bread, Pizza Crust, Noodles	RS2, RS3	Improved texture, increased dietary fiber content, better nutritional profile, lower risk of chronic degenerative diseases, low glycemic index, and improved gut health.	[222,243,244]
Confectionery	Chocolate, Candy Bars	RS2, RS4	Reduced sugar content, lower glycemic index, rich in fiber, high in antioxidants.	[245,246]

## Data Availability

The original contributions presented in the study are included in the article, further inquiries can be directed to the corresponding author.
